# Supplementary material for: Co-Expression of Immunohistochemical Markers MRP2, CXCR4, and PD-L1 in Gallbladder Tumors Is Associated with Prolonged Patient Survival
Source: Cancers (Basel). 2023 Jun 30;15(13):3440. doi: 10.3390/cancers15133440 (PMC10340206; doi:10.3390/cancers15133440)
Supplement: Supplementary file 1 [file cancers-15-03440-s001.zip › cancers-2445727-supplementary.pdf]

cancers

MDPI

*Supplementary Materials*

# Co-Expression of Immunohistochemical Markers MRP2, CXCR4, and PD-L1 in Gallbladder Tumors Is Associated with Prolonged Patient Survival

**Andrés Tittarelli, Omar Barría, Evy Sanders, Anna Bergqvist, Daniel Uribe Brange, Mabel Vidal María Alejandra Gleisner, Jorge Ramón Vergara, Ignacio Niechi, Iván Flores, Cristián Pereda, Cristian Carrasco, Claudia Quezada-Monrás and Flavio Salazar-Onfray**

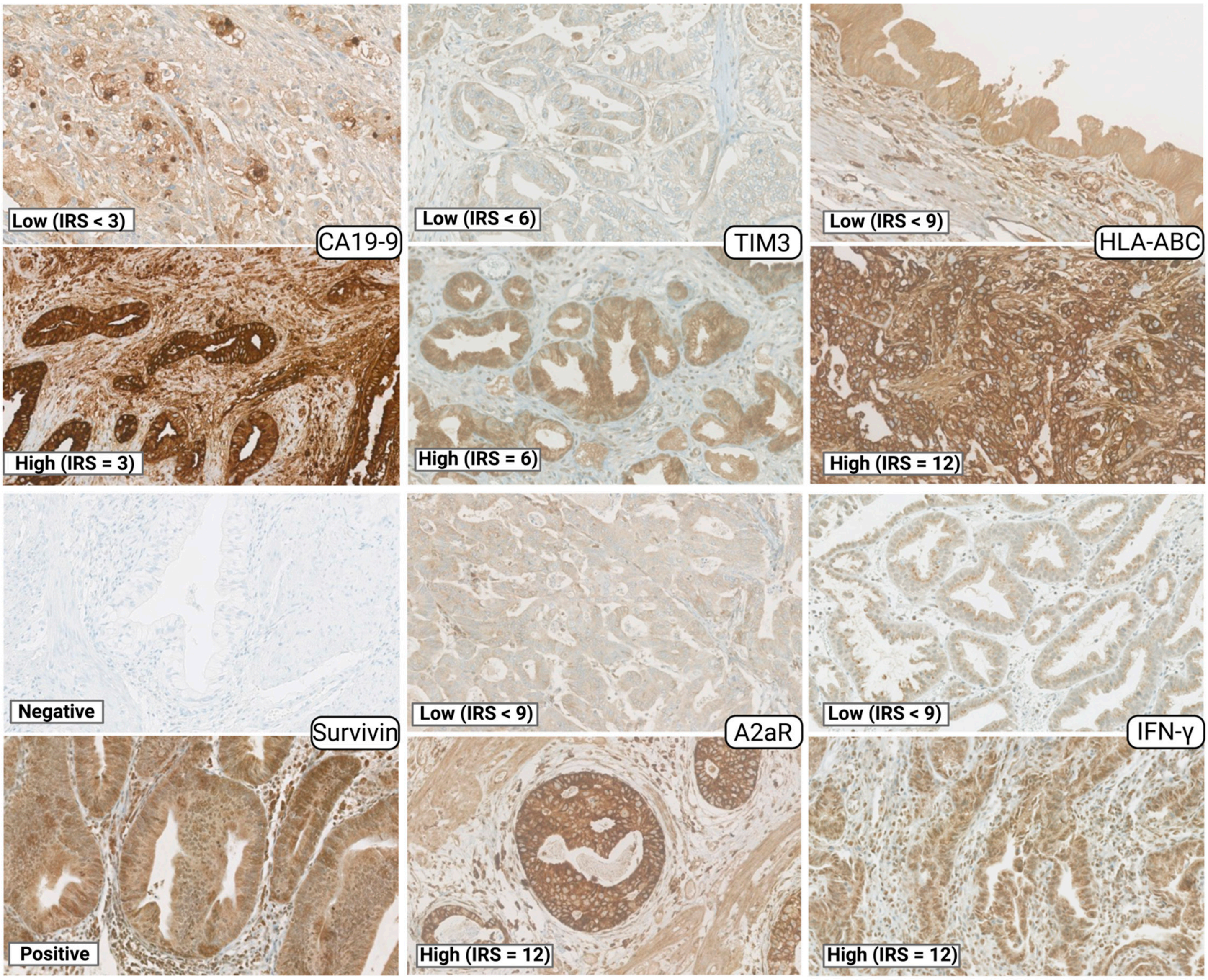

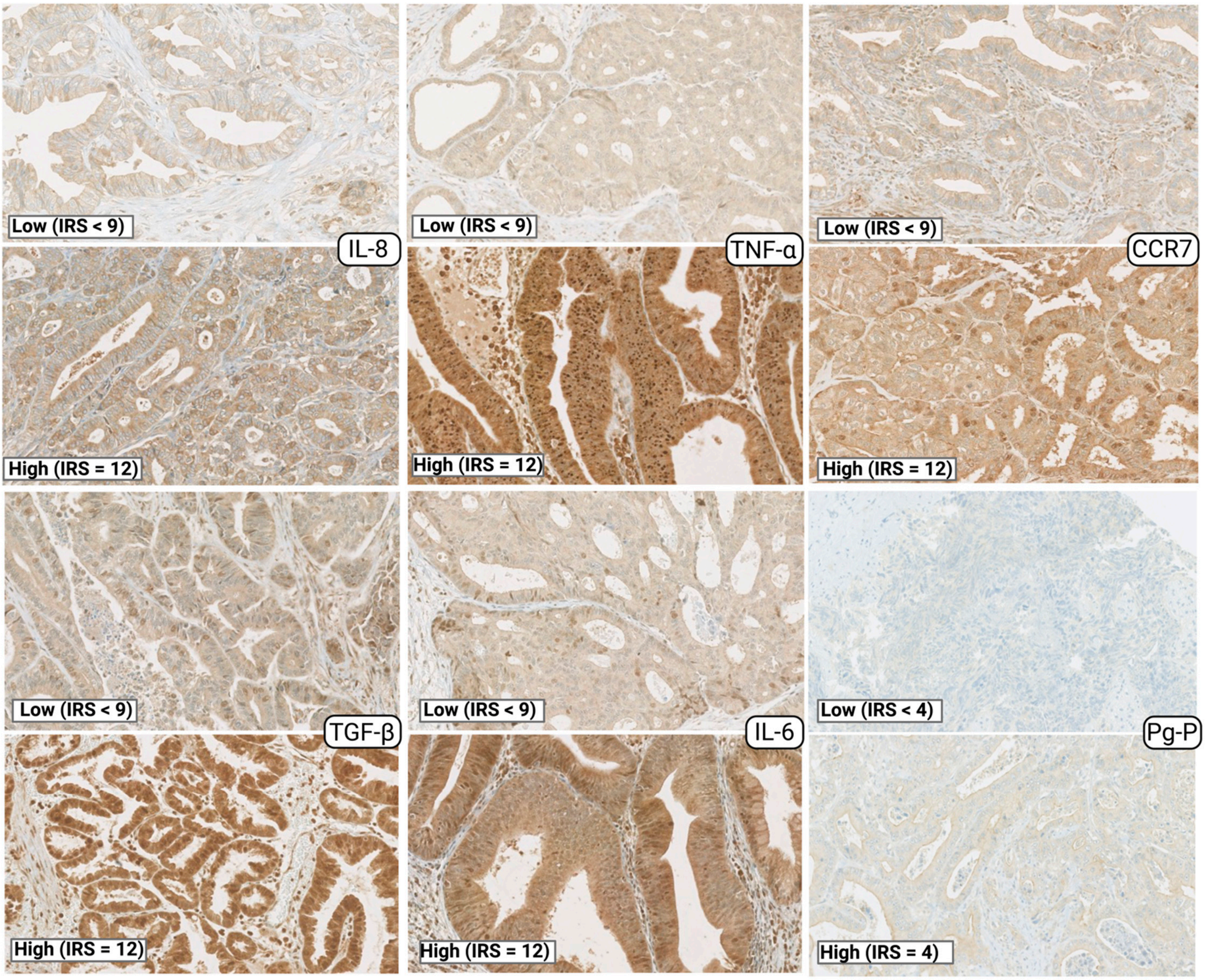


**Figure S1.** Examples of immunohistochemical images for representative tumor markers. Images correspond to 80X magnifications of microarray photographs. IRS, immunoreactive score.

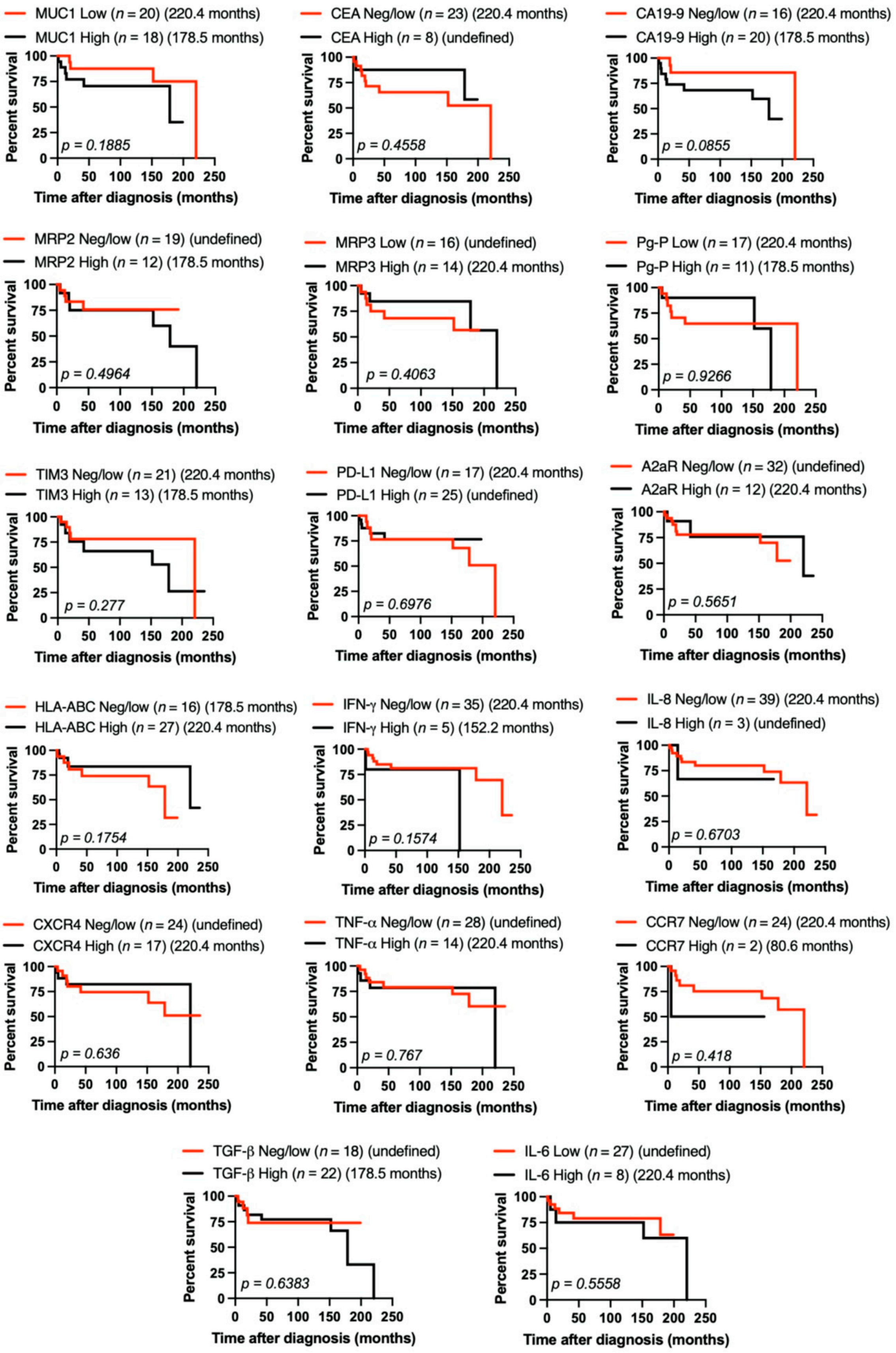


**Figure S2.** Association among tumor markers and overall survival (OS) of gallbladder cancer (GBC) patients with early-stage primary tumors. Kaplan–Meier post-diagnosis OS estimation of early-stage tumor-bearing GBC patients, according to tumor expression patterns (neg/low vs. high expression). Each graph shows the number of patients in each group (*n*) and the median OS time in months. The *p*-values were calculated using a log-rank (Mantel–Cox) test.
